# Are dentists aware of post-traumatic trigeminal neuropathic pain? A web-based epidemiological survey

**DOI:** 10.22514/jofph.2025.009

**Published:** 2025-03-12

**Authors:** Domenico Viscuso, Marco Storari, Cinzia Casu, Alessandra Scano, Eleonora Aru, Germano Orrù, Valentino Garau

**Affiliations:** ^1^Department of Surgical Science, College of Dentistry, University of Cagliari, 09124 Cagliari, Italy; ^2^Department of Surgical Science, Oral Biotechnology Lab (OBL), University of Cagliari, 09124 Cagliari, Italy

**Keywords:** Dentoalveolar pain, Anaesthesia dolorosa, Painful post-traumatic trigeminal neuropathy, Web epidemiological survey, Preventive medicine

## Abstract

**Background**: Post-traumatic trigeminal neuropathic pain represents neuropathic pain in the distribution of the trigeminal nerve caused by trauma to the trigeminal nerve. Dental traumatic interventions, such as root-canal therapy and extractions, are reported to precede, in some cases, the onset of the disease. The current study aims to investigate how much dentists are trained to recognize, treat or properly address patients suffering from Post-traumatic trigeminal neuropathic pain. **Methods**: Data were collected from a large sample of Italian dentists in 2021. The setting of this study relates to an epidemiological survey conducted on the web. Google Forms, a product of Google Inc., was used as the operating system. An online questionnaire was sent to each participant, and the degree of knowledge of the disease along with the direct experience of having encountered it was investigated through specific multiple-choice questions. **Results**: 634 dentists participated in the survey. 29% of participants declared to be unaware of the existence of Post-traumatic trigeminal neuropathic pain. 70% of dentists reported to have had patients suspicious of such pain in their clinical activity, following endodontic treatment (60%), tooth extraction (43%), spontaneously (37%) or other dental therapies (21%). When encountered, only in one out of three cases were patients sent to a pain specialist, and in most cases dentists performed irreversible therapies the site of the pain. **Conclusions**: This study evidences a major public health problem, such as the incapability of clinicians to perform a correct diagnosis and management of Post-traumatic trigeminal neuropathic pain. Such a lack of knowledge costs the patients mistaken and irreversible surgical therapies in many cases, and resulting delays in receiving proper diagnosis and management that could affect the success of the treatment. Furthermore, the unawareness had high socioeconomic costs for both the healthcare system and the patients due to the disability. **Clinical Trial Registration**: NP/2021/5460, Institutional Review Boards of the University of Cagliari, Italy.

## 1. Introduction

Post-traumatic trigeminal neuropathic pain (PTTN), also called anaesthesia dolorosa, or painful post-traumatic trigeminal neuropathy, is a disorder belonging to the wider family of neuropathic pain. As such, it does not derive from peripheral stimuli but is due to a dysfunction or a lesion of the nervous system itself [1]. PTTN is defined as unilateral or bilateral oral and/or facial pain whose onset follows a trauma to the trigeminal nerve associated to other signs and symptoms of trigeminal dysfunction, and pain persists or recurs for more than three months [2]. Pain generally arises as the result of traumatic interventions in the trigeminal system, such as root-canal treatments and extractions. Patients most often report an intraoral, localized, aching, heavy and continuous pain with moderate intensity in a tooth, in the site where a tooth was previously present, or in surrounding tissues [3]. This last aspect should be pivotal to differentiate between Persistent Idiopathic Dentoalveolar Pain (PIDP) and PTTN, among the most challenging diseases to diagnose and treat in the orofacial pain specialty. Indeed, both PTTN and PIDP, also called “phantom-tooth pain”, share many clinical characteristics in terms of frequency of pain (from constant to episodic), quality of pain (burning, sharp, shooting, throbbing, dull aching and crushing) and localization of pain (unilateral, intraoral; PTTN, differently to PIDP can be rarely extraoral) [4]. Historically, considerable heterogeneity has existed in the terminology used to indicate orofacial neuropathic pain. The terms phantom tooth pain and atypical facial pain, starting from the 1970s, and subsequently followed by atypical odontalgia, have been used for years indiscriminately to refer to conditions that only now we know are clearly distinct [5, 6, 7, 8]. The recent International Classification of Orofacial Pain (ICOP) has addressed the implicit ambiguity in the terminology by abandoning such terms and precisely separating the various forms of orofacial neuropathic pain. PTTN occurs in 3% to 6% of patients who underwent endodontic treatment, with a higher prevalence in the female gender and after the 4th–5th decade of age [3, 9]. The maxillary molar and premolar regions are mostly affected [10]. By contrast, other authors reported a prevalence of PTTN after successful root canal treatment of 12% [11]. Additionally, the presence and duration of at least 3 months of any preoperative pain, any previous complaint of chronic pain in any part of the body, and previous painful treatment in the orofacial region are other risk factors for developing PTTN [3]. Depression, anxiety, psychotic, and bipolar disorders are also listed as factors associated with PTTN [12]. The exact mechanisms leading to PTTN has not yet fully understood but a complex cascade of both peripheral and central nervous system events is most likely the major responsible of neural dysfunction [13]. Despite the growing evidence, no therapeutic approaches have been yet standardized in the treatment of PTTN. The efficacy of systemic drugs, such as anticonvulsants, local anesthetics and antidepressants, is known in targeting neuropathic pain [14, 15]; however, due to the important side effects of systemic drugs, topical therapy often represents a prevalent modality in pain management [16, 17]. Regarding the intraoral delivery of topical products, to address the problem of washing off by the saliva and the subsequent dispersion from the affected area of the substance, some authors proposed the use of a silicon stent [18]. Lastly, surgical techniques may represent valid options [19], while further studies are needed to evaluate onabotulinum toxin’s efficacy [20]. Nowadays, clinicians are not trained and, furthermore, not able to recognize, treat, or know how to properly address patients suffering from PTTN [12].

In the present situation, the purpose of this study is to investigate the level of knowledge of PTTN among Italian dentists and to raise awareness about this important public health problem. To accomplish the aim, the authors provided an online questionnaire entitled “University of Cagliari: Epidemiological Study on the Level of Knowledge of Italian Dentists About Post-traumatic trigeminal neuropathic pain”.

## 2. Materials and methods

The current prospective cohort study was conducted in accordance with the Declaration of Helsinki, and it was approved by the Institutional Review Boards at the University of Cagliari with the referent code PROT. NP/2021/5460.

### 2.1 Study population

Data were collected from a large sample of Italian dentists in 2021. The eligibility period was established from October to December 2021. Those who decided to participate could get access to the online survey directly via social media. The study was conducted completely blindly, as the authors received only nameless answers. In every part of the study, personal data was not attributable to the participants, thanks to the operating system used. However, individuals were asked to provide anonymous, informed consent prior to submission.

### 2.2 Procedure

The setting of this study relates to an epidemiological survey conducted on the web. Google Forms, a product of Google Inc., was used as the operating system as it is a free and publicly accessible online portal that allows users to easily interact with interviewers. To achieve this goal, the authors provided an online questionnaire entitled “University of Cagliari: Epidemiological Study on the Level of Knowledge of Italian Dentists About Post-traumatic trigeminal neuropathic pain”. The questionnaire was combined with a short introductory letter. The survey took approximately 3 minutes to complete in all its parts. The questionnaire consisted of several multiple-choice questions regarding different clinical aspects related to PTTN and the degree of experience of clinicians with the disorder (Table 1).

**Table 1.** Questionnaire.

Topic	Questions	Answers
	(1) Do you claim to be a dentist?	Yes
Knowledge of PTTN		
	(2) Are you aware of the existence of the PTTN?	Yes; No
	(3) Have you ever received teachings about PTTN during your undergraduate or postgraduate university studies?	Yes; No
Clinical experience with PTTN		
	(4) Are you aware of the need to perform a differential diagnosis between orofacial neuropathic pain and primary odontogenic pain (of dental or periodontal origin)?	Yes; No
	(5) Have you ever had patients with PTTN in your professional activity?	Yes; No
	(6) Have you ever sent a patient to a pain specialist because of a suspected diagnosis of neuropathic pain in the presence of pain “reported” by the patient in a tooth and/or in the periodontium?	Yes; No
	(7) If yes, the pain arose: (You can give more than one answer. If the answer is “it’s never happened to me”, then don’t answer.)	Spontaneously; Following a well-performed root canal therapy; Following a well-performed extraction; Following other dental therapies
	(8) If you had experience with patients with PTTN; following a well-performed root canal therapy, how did you manage the problem? (You can put more than one answer; If your response is “it has never happened to me”, then please refrain from answering further.)	You undertook root canal retreatment; You performed an apicectomy; You treated or extracted neighboring teeth, thinking it was dental pain; You treated the patient pharmacologically; You referred the patient to a pain specialist; You extracted the tooth; You didn’t know what to do
	(9) If you had experience with patients with PTTN following a well-performed tooth extraction, how did you manage the problem? (You can give more than one answer. If the answer is “it has never happened to me”, then do not answer.)	You undertook a surgical reevaluation; You treated or extracted neighboring teeth, thinking of dental pain; You pharmacologically treated the patient; You sent the patient to a pain specialist; You didn’t know what to do
BACPrivacy	(10) I accept the processing of my data pursuant to Legislative Decree. n.101/2018.	Yes

*PTTN: Post-traumatic trigeminal neuropathic pain.*

### 2.3 Variables

To be eligible to participate in the study, one must have the title of dentist. The first question of the questionnaire asked, in fact, “Do you claim to be a dentist?”.

The degree of knowledge of PTTN was investigated below through specific multiple-choice questions, some ordinal and some nominal. Firstly, participants were asked, “Are you aware of the existence of the PTTN?” (Yes, No). Subsequently, the level of education and teachings received by participants about PTTN was assessed through the question, “Have you ever received teachings about PTTN during your undergraduate or postgraduate university studies?” (Yes, No).

The second part of the questionnaire aimed to evaluate the direct clinical experience of dentists with the PTTN through a sequence of questions related to their daily clinical practice. Firstly, the self-assessment capability of dentists in evaluating their approach to PTTN was investigated with the question “Are you aware of the need to perform a differential diagnosis between orofacial neuropathic pain and primary odontogenic pain (of dental or periodontal origin)?” (Yes, No). Whether or not participants had encountered a suspected PTTN in their working experience was ascertained with the following question: “Have you ever had patients with PTTN in your professional activity?”. The question “Have you ever sent a patient to a pain specialist because of a suspected diagnosis of neuropathic pain in the presence of pain “reported” by the patient in a tooth and/or in the periodontium?” (Yes, No) was asked to test the ability to recognize pain of a suspected non-odontogenic nature. If the clinician has had experience with patients with suspected neuropathic pain, then he was asked to indicate the procedure(s) following which the onset of the symptomatology was reported: “If yes, the pain arose: (You can give more than one answer. If the answer is “it’s never happened to me,” then don’t answer.) (Spontaneously, following a well-performed root canal therapy, following a well-performed extraction and following other dental therapies.) The therapeutic approach adopted following the suspicion of neuropathic pain was evaluated through two other questions; the former was “If you had experience with patients with PTTN following a well-performed root canal therapy, how did you manage the problem? (You can put more than one answer. If the answer is “it has never happened to me”, then do not answer). You undertook root canal retreatment, you performed an apicectomy, you treated or extracted neighboring teeth, thinking it was dental pain. You treated the patient pharmacologically. You referred the patient to a pain specialist. You extracted the tooth; you didn’t know what to do. The latter question was “If you had experience with patients with PTTN following a well-performed tooth extraction, how did you manage the problem? (You can give more than one answer. If the answer is “it has never happened to me,” then do not answer.” You undertook a surgical reevaluation, you treated or extracted neighboring teeth, thinking of dental pain, you pharmacologically treated the patient; you sent the patient to a pain specialist; you didn’t know what to do).

### 2.4 Statistical analysis

The collected data were organized in Excel worksheets. The aim was an exploratory analysis to understand what kinds of data were collected and how they were distributed. The Python language was used for statistical analysis, exploratory analysis, and correlation studies through the packages Pandas and SciPy (version 2.2.3, Python Software Foundation©, Fredericksburg, VA, USA). To carry out the association analysis between the questions present in the questionnaire, two methods were used: the chi-square test and Fisher’s exact test. In all the analyses, the null hypothesis was that there was no association between the two variables considered. 0.05 was used as the alpha threshold, therefore a maximum error of 5% was tolerated. Subsequently, the answers were filtered between those in which the pain appeared following a root canal treatment or extraction; the answers to the question “If you have had patients with PPTN that arose after a well-performed root canal therapy/tooth extraction, how did you manage the problem?” were transformed into binary variables, so if at least one of the answers selected by the participants was that they sent the patient to a pain specialist, “Yes” was entered as the answer (1), otherwise “No” was chosen to refer to all the other cases (0).

## 3. Results

### 3.1 Baseline characteristics

634 dentists participated to the survey. Fig. 1 displays the baseline characteristics of the sample; 71.3% of the participants declared that they knew the existence of the PTTN, while 28.7% declared that they were unaware of it. Furthermore, almost one out of five participants declared that they had not received any teachings about the PTTN.

**Fig. 1.** Distribution of respondents’ baseline characteristics. PTTN: Post-traumatic trigeminal neuropathic pain. Blue is referred to ”Yes”, red to ”No”.

### 3.2 Clinical experience

Data about the clinical experience of clinicians with PTTN are collected in Fig. 2. 88.3% of the participants stated that they were aware of the need to perform a differential diagnosis between orofacial neuropathic pain and primary odontogenic pain, whether of dental or periodontal origin. We asked for the capability of referring patients: 49% of participants reported having referred patients with a suspected diagnosis of neuropathic pain to a pain specialist. By contrast, it emerged that 51% of participants reported that they had not sought referral from a neuropathic pain specialist in their clinical practice. By the way, it also emerged that 71.7% of Italian dentists had encountered cases of suspected PTTN in their clinical practice.

**Fig. 2.** Participants’ clinical experience with PTTN. PTTN: Post-traumatic trigeminal neuropathic pain. Blue is referred to ”Yes”, red to ”No”.

### 3.3 Causes of PTTN

412 participants defined the triggering event that caused neuropathic pain (Fig. 3). Participants had the possibility of listing more than one cause. Among them, 37.10% reported that in their experience PTTN arose spontaneously without any dental procedures that triggered it. The rest of the participants, instead, recognised that PTTN manifested following a triggering cause. Higher percentages emerged when root canal treatments and extractions were taken into consideration. Root canal therapy emerged as the dental procedure most frequently recognized as a triggering event for PTTN. Indeed, well-performed root canal therapy was identified as responsible in 60.7%, while well-executed extractions in 42.7%. Furthermore, dental procedures other than well-performed root canal therapy and extractions were found to be causative factors for PTTN in 21.4% of cases.

**Fig. 3.** Triggering event for PTTN reported by dentists. PTTN: Post-traumatic trigeminal neuropathic pain.

### 3.4 Clinicians’ approach to PTTN

370 clinicians positively answered when asked to approach PTTN following a well-performed root canal therapy. Fig. 4 shows that in most cases, namely 57.6%, participants reported that they treated the patient pharmacologically. On the other hand, in a smaller percentage (48.6%), participants declared that they performed an endodontic retreatment in the attempt to resolve the PTTN. In more than one out of five cases, *i.e.*, 22.4%, participants declared that they extracted the previously devitalized tooth, while in 8.6% of cases, an apicectomy was performed. In a very similar percentage, participants reported that they treated or extracted teeth close to the ones originally treated endodontically. In less than one out of three cases, dentists declared to have referred the patient to a pain specialist for the evaluation, and even in 13.8% of the cases, they reported not knowing how to proceed (Fig. 4).

**Fig. 4.** Distribution of different dentists’ approaches to a suspicion of PTTN after a well-performed root canal therapy. PTTN: Post-traumatic trigeminal neuropathic pain.

When a well-performed extraction was taken into account as a triggering factor for PTTN (Fig. 5), 339 replies were collected. In the majority of cases, namely 70.8%, participants stated that they treated the patient pharmacologically, while in one out of four cases, they undertook a surgical revision of the post-extraction site. In lesser percentages, participants reported having treated or extracted neighboring teeth (4.10%), and in 11.4% of the cases, they admitted not knowing how to proceed. Similarly, to what happened for PTTN following root canal therapy, 30.7% of participants reported that they referred the patient to a pain specialist for evaluation.

**Fig. 5.** Distribution of different dentists’ approaches to a suspicion of PTTN after a well-performed dental extraction. PTTN: Post-traumatic trigeminal neuropathic pain.

The inferential analysis was conducted in order to see potential significant associations among the answers within the questionnaire. Questions from two to six were firstly considered, and for each possible combination the association was evaluated through both the chi-square test (Table 2) and Fisher’s exact test (Table 3). The two tests provided congruent results for each combination. Participants who weren’t aware of the existence of PTTN demonstrated a statistical significance (*p * < 0.05) in responding negatively to all the other questions taken individually into exam. Similarly, those who were not aware of the need for a differential diagnosis between primary neuropathic pain and odontogenic pain showed a significant positive association (*p * < 0.05) with not being aware of PTTN, having never received teachings about it, having never met patients with PTTN or having never referred patients with a suspected diagnosis of PTTN to a specialist.

**Table 2.** Chi-square test. To carry out the association analysis between the questions present in the questionnaire (*p*. value).

	Question 2	Question 3	Question 4	Question 5	Question 6
Question 2					
Question 3	**2.45 × 10^−12^**				
Question 4	**2.09 × 10^−10^**	**<0.001**			
Question 5	**<0.001**	0.08	**8.08 × 10^−7^**		
Question 6	**3.49 × 10^−8^**	0.15	**0.02**	**5.29 × 10^−12^**	

*Bolded data are those data which are statistically significant.*

**Table 3.** Fisher’s exact test. To carry out the association analysis between the questions present in the questionnaire (*p*. value).

	Question 2	Question 3	Question 4	Question 5	Question 6
Question 2					
Question 3	**1.22 × 10^−15^**				
Question 4	**1.71 × 10^−9^**	**<0.001**			
Question 5	**<0.001**	0.08	**5.91 × 10^−7^**		
Question 6	**7.65 × 10^−8^**	0.18	**0.02**	**4.43 × 10^−12^**	

*Bolded data are those data which are statistically significant.*

When dentists’ awareness was filtered focusing on how they approached potential PTTN arisen following a root canal treatment or tooth extraction, only those who were aware of PTTN existence sent the patient to a pain specialist. However, the referral happened only in case of pain appeared following a well-performed root canal (*p* = 0.003).

## 4. Discussion

The current study aims to report the level of knowledge of dentists about the diagnosis and management of PTTN. Web-based epidemiological survey’s data from a large Italian sample were collected. In the first part of the questionnaire, awareness related to the PTTN was assessed, and it was demonstrated that one out of three dentists didn’t even know of the existence of such a disease. This first evidence highlights a major public health problem already reported, such as the incapability of clinicians to perform a correct diagnosis and management, or at worst, to mistake both diagnosis and treatment [21, 22]. Specifically, it was found that patients with PTTN often suffer a diagnostic delay of more than 2 years prior to receive a correct diagnosis [23]. Similarly, when other subtypes of chronic pain were taken into consideration, the lack of dentists’ ability to recognize and properly manage or refer the disease created considerable delays for patients to be correctly addressed [24]. In such scenario, the cause probably should be investigated at educational levels. In fact, we found that more than 80% of dentists did not receive any teaching about PTTN during the course of their studies.

In the second part of the questionnaire, the survey focused on the clinical experience of dentists with PTTN. Although there was scarce knowledge, more than 70% of dentists said to have encountered a suspected case of PTTN in their clinical practice. Such data should not be surprising in light of its high prevalence. It was in fact demonstrated that three to six well-performed endodontic therapies lead to the onset of PTTN. Similarly, Nixdorf *et al*. [25] estimated that 3.4% of patients who underwent canal therapy developed PTTN subsequently. As expected, it also emerged in our study that the most common possible cause of a suspected PTTN was root canal treatment. In six out of ten cases, the participants related the development of the disease in their clinical experience to well-performed root canal therapy. If we apply such percentages to the number of root canal therapies performed worldwide [26], it appears that more than 150,000,000 individuals are at risk of developing PTTN. In the present picture, the only partially reassuring aspect that emerges from the current study is that, in the event of pain arising following a well-performed root canal, dentists who were aware of PTTN had diagnostic suspicions and referred the patient to a specialist. The association between the onset of PTTN and root canal therapy could be explained by pathophysiological changes leading to the development of chronic pain that would occur in specific circumstances that are not yet well understood. It is also assumed that PTTN may be present from the outset and that endodontic therapy is consequently performed due to the incorrect diagnosis with the intention of relieving symptoms [27].

A main consequence of the lack of differential diagnosis is the fact that the onset of PTTN is generally accompanied by ineffective, unnecessary and irreversible dental procedures (root canal therapy, apicectomy, extraction) of the tooth from which it is wrongly suspected that the pain arises, or even of the sided teeth [28]. However, this represents a mere and not justified attempt to end the patient’s pain. A very similar analysis occurred when dental extractions were taken into account, as almost half of dentists reported to have experienced a suspicion of PTTN after a well-executed dental extraction.

The inferential statistics demonstrated that unawareness brought unawareness. The lack of information about PTTN significantly correlated with the inability to recognize it. Dentists who declared the less education about PTTN emerged more prone to respond that they never met it during their professional lifespan and to not acknowledge the necessity of a differential diagnosis between odontogenic and neuropathic toothache.

The study also analyzed the behavior of dentists when suspicion of PTTN arose after well-performed dental procedures. The majority of participants established drug-based therapy for pain control in cases of PTTN developed after endodontic treatment and dental extraction, respectively, in 58% and 71% of cases. A root canal retreatment was attempted in almost half of cases, and a more invasive apicectomy was performed in one out of ten cases when root canal treatment was the reported triggering event. Only in a few cases the adjacent teeth were treated. Similarly, in only 4% of cases, nearby teeth were endodontically treated or extracted, when instead tooth extraction was reported to be the causative factor, while in 26% of cases a surgical review of the post-extractive site was carried out in the attempt to regress the symptomatology. Furthermore, dentists appeared to not know what to do in cases of suspicion of PTTN in 11 to 14% of all cases. Unfortunately, only one out of three dentists referred the patient to pain specialists in the case of suspected PTTN.

In the current study, we didn’t investigate the dentist’s habit to evaluate the patient’s psychologic state during the interview. However, such behavior may be useful, as patients with PTTN were demonstrated to have a higher frequency of psychiatric disorders, especially depression and somatization disorders [12] and a very reduced quality of life [29]. Another limitation of the study is the fact that respondents were drawn from one state, *i.e.*, Italy, therefore the results may not be generalized to other countries. Further studies should focus on the clinical patterns of practice among dentists, as well as reinforcing and enabling factors that influence them to recognize and/or make recommendations for patients with potential PTTN.

In light of such confusion, Malacarne A *et al*. [30] further confirmed the need for increasing knowledge about PTTN epidemiology, diagnosis and management to solve an emerging public health concern. To date, very few data exist on estimated costs to patients, healthcare systems and society due to PTTN. For the first time, a recent Belgian study pointed out a median annual direct cost of €1396 per patient over a period of 5 years after PTTN diagnosis [31]. If considering neuropathic pain without differentiation in its forms, annual direct costs per patient range from €1939 in Italy to €3131 in Spain [32]. However, the major burden is represented by the indirect costs which are mainly driven by the productivity loss and varied from €9305 in Italy to €14,446 in Germany per patient [32].

A few considerations should be outlined in conclusion. Cooperation of dentists between each other and with physicians is probably not adequate as referring patients emerged to be a not common practice. Similarly, their education, including dental school education and postgraduate training, is deficient. In this sense, universities and public health systems should work together to fill the gap as PTTN, but more generally orofacial pain, still remains a severe inadequacy of the healthcare system, and consequently a great burden for the general population, in Italy as in other countries such as USA [33]. In Italy, the law no. 38/2010, the very first in Europe, which is highly innovative and a point of excellence in our country, guarantees access to pain therapy and palliative care in order to ensure respect for human dignity and autonomy. Furthermore, it defines the importance to raise awareness among health workers to detect and evaluate pain during clinical visits and to consider pain not only a symptom, as it could be. Formally the law exists but its application is lacking, as we can observe by the results of the current study. Therefore, our proposal to address the issue is structured into three levels: (1) the promotion of awareness campaigns dedicated to the education of both physicians and general population, (2) the strengthening of both undergraduation and postgraduation specific programs to provide at least basic knowledge on orofacial pain, and (3) introduce orofacial pain specialists in both dental school’s departments and hospitals.

## 5. Conclusions

The current study highlighted the lack of knowledge of dentists about PTTN, although most of them encountered it in their clinical activity during their lifespan. Given the current situation, it is crucial to intensify efforts in teaching and raising awareness about PTTN, a prevent disease often resulting from routine dental procedures. Due to the high socio-economic costs of PTTN, healthcare systems and universities should cooperate to facilitate patients’ access to adequate diagnosis and therapies.

## 6. Key findings

Although the majority of dentists encountered PTTN during their professional careers, the investigation brought to light their lack of feeling about this clinical approach. As PTTN appears to be a fairly common condition that develops after normal dental treatments, more efforts should be made to increase training and knowledge about it in the current context. In this context, the present investigation wants to incite the dentist to become more aware of this aspect.

## 7. Limitations

In the current study, we didn’t investigate the dentist’s habit to evaluate the patient’s psychologic state during the interview. However, such behavior may be useful, as patients with PTTN were demonstrated to have a higher frequency of psychiatric disorders, especially depression and somatization disorders [12] and a very reduced quality of life [29]. Another limitation of the study is the fact that respondents were drawn from one state, *i.e.*, Italy, therefore the results may not be generalized to other countries. Further studies should focus on the clinical patterns of practice among dentists, as well as reinforcing and enabling factors that influence them to recognize and/or make recommendations for patients with potential PTTN.
